# Supplementary figures and images for: Transmission potential of mpox in mainland China, June-July 2023: estimating reproduction number during the initial phase of the epidemic
Source: PeerJ. 2024 Feb 8;12:e16908. doi: 10.7717/peerj.16908 (PMC10859083; doi:10.7717/peerj.16908)

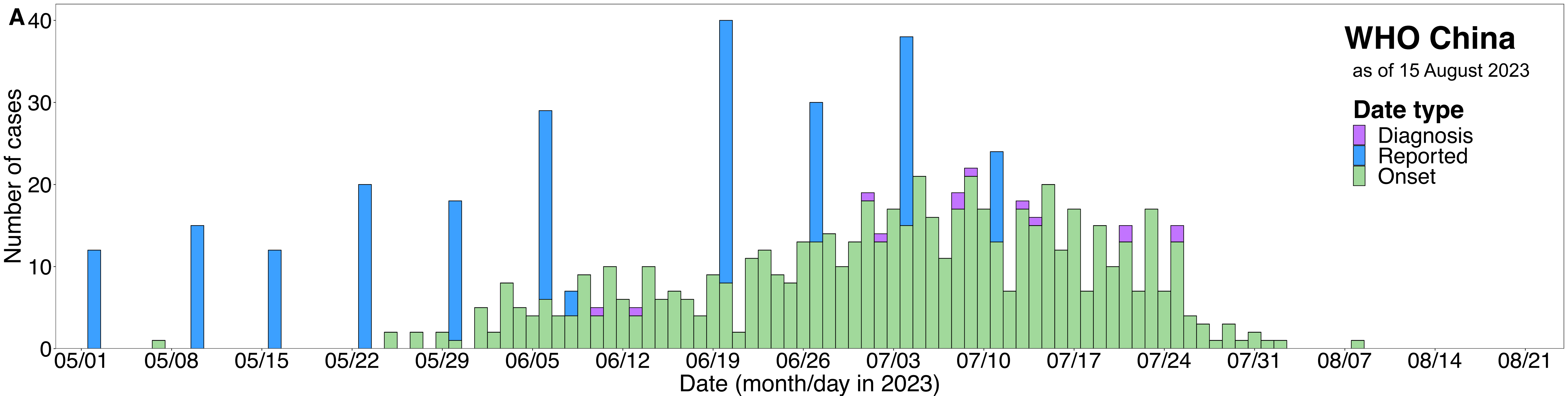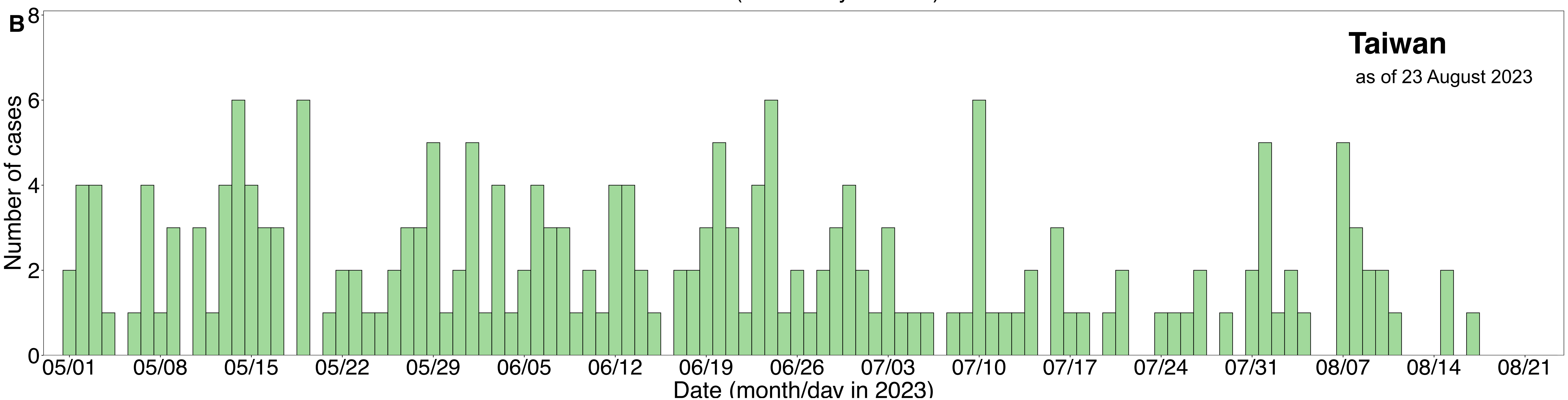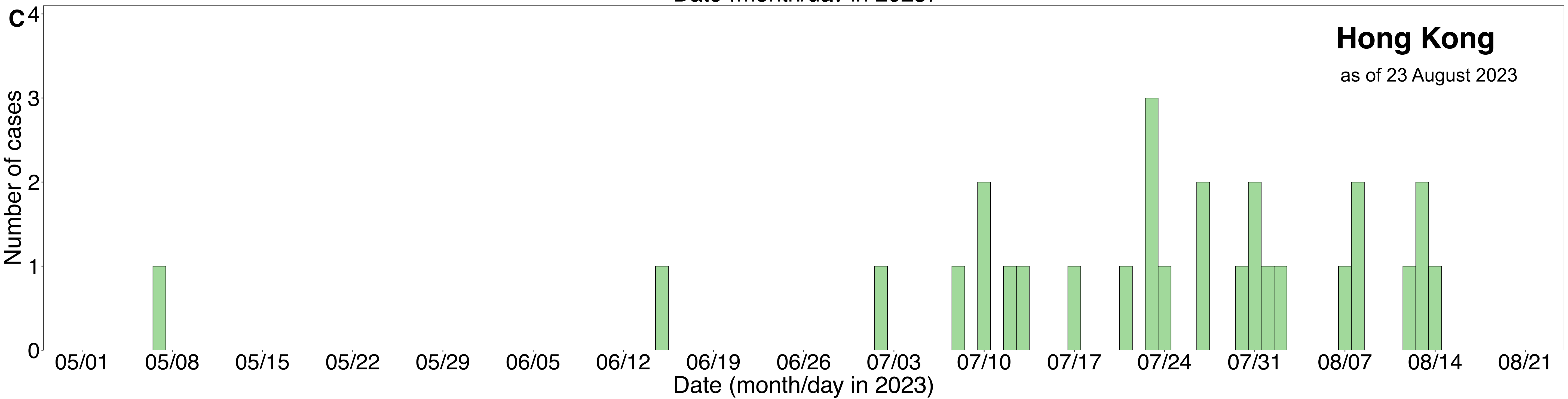

Supplement: Supplemental Information 1 — The snapshot dates are indicated in the legends. Each case count is subtyped by the date of symptom onset, the date of diagnosis, and the date of reporting. [file peerj-12-16908-s001.pdf]

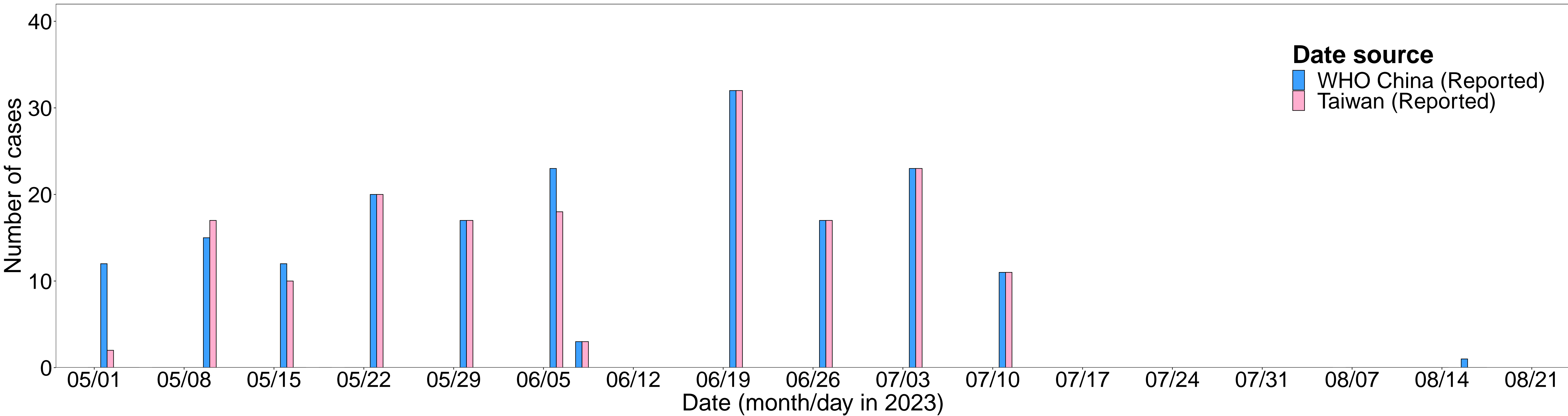

Supplement: Supplemental Information 2 — The dates of reporting for Taiwan were aggregated to the dates of reporting from the WHO dataset and Taiwan cases beyond the latest 11 July 2023 were omitted. [file peerj-12-16908-s002.pdf]

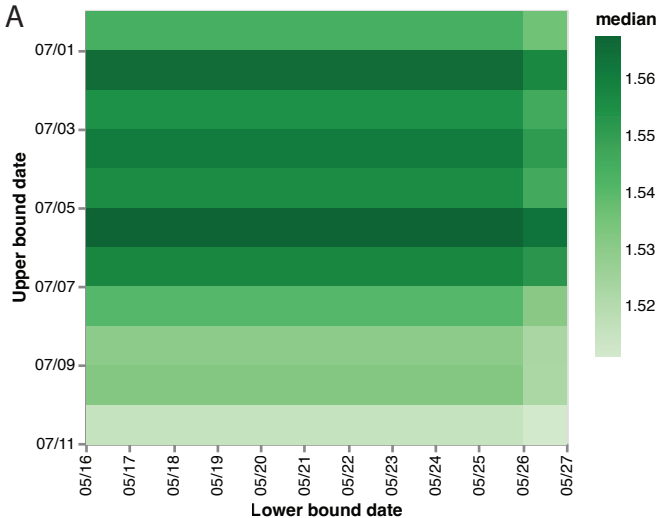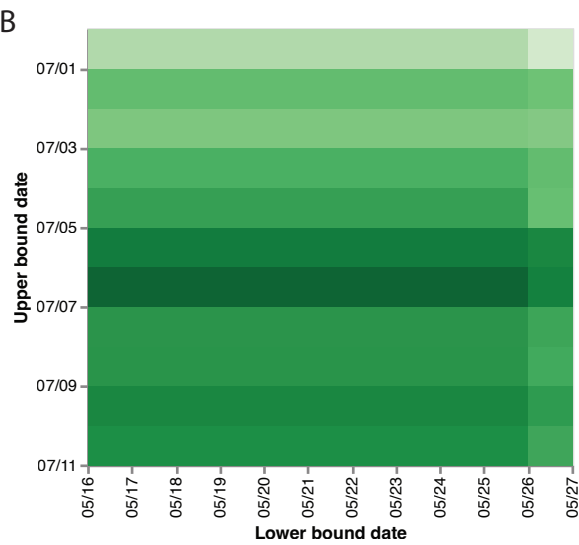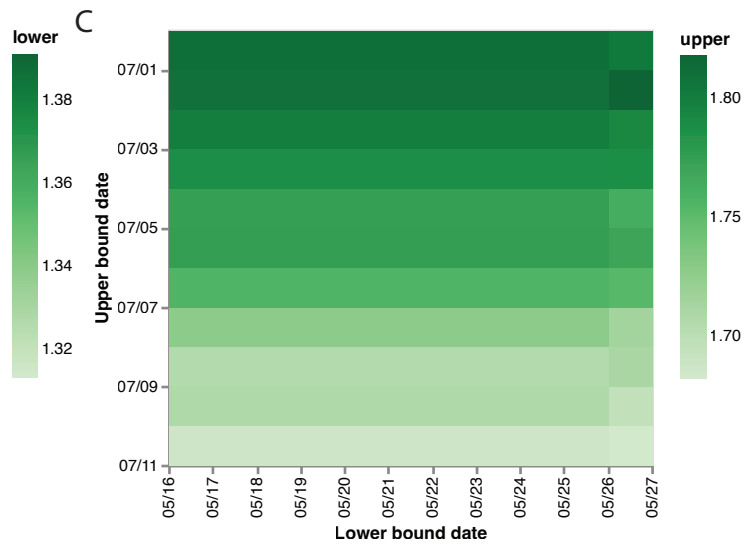

Supplement: Supplemental Information 3 — (A) shows the posterior median of Re, (B) and (C) show the lower (2.5th percentile) and upper (97.5th percentile) bound of the 95% credible interval, respectively. Horizontal axis indicates the lower bound, while the vertical axis indicates the upper bound which were used for the exponential phase window. The date is indicated as month/day in 2023. [file peerj-12-16908-s003.pdf]

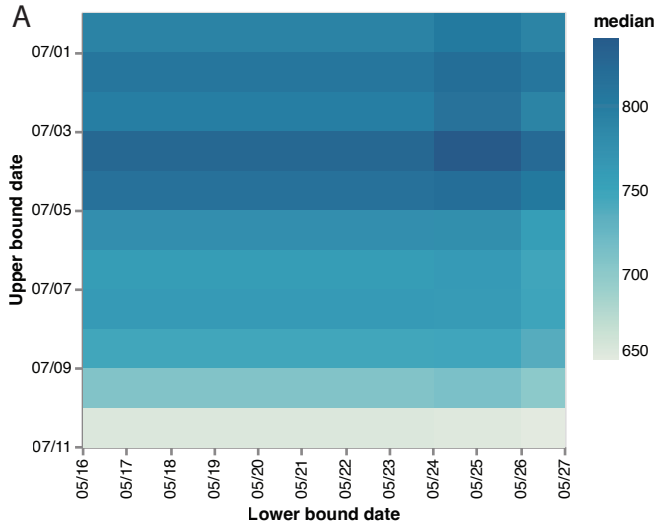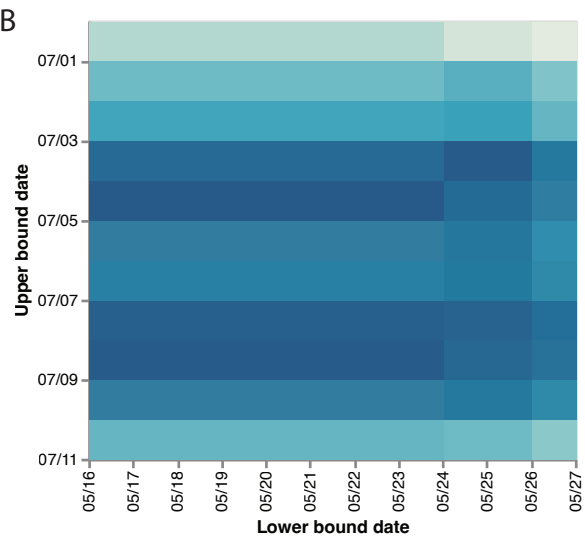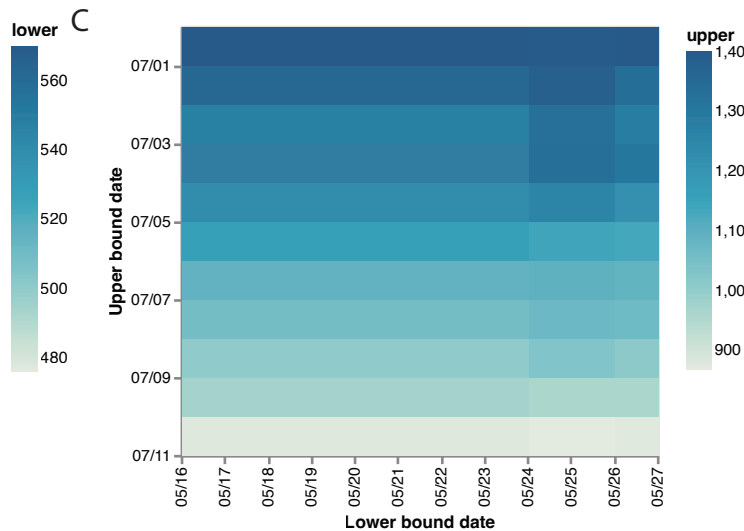

Supplement: Supplemental Information 4 — (A) the posterior median of number of cases, (B) and (C) the lower (2.5th percentile) and upper (97.5th percentile) bound of the 95% credible interval, respectively. Horizontal axis indicates the lower bound date, while the vertical axis indicates the upper bound which were used for the exponential phase window. The date is indicated as month/day in 2023. [file peerj-12-16908-s004.pdf]

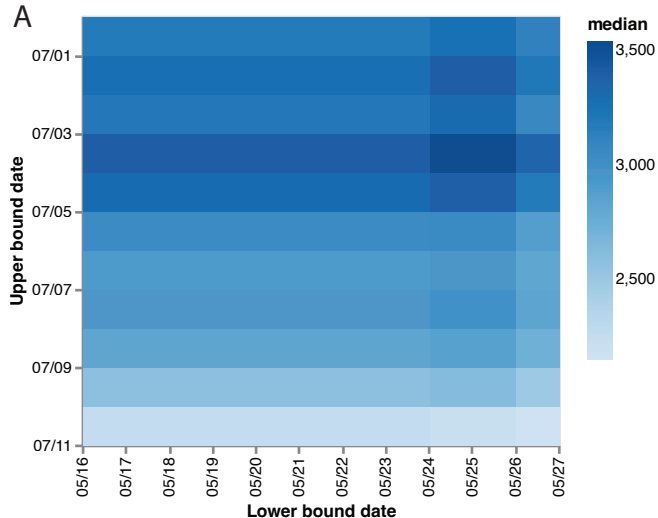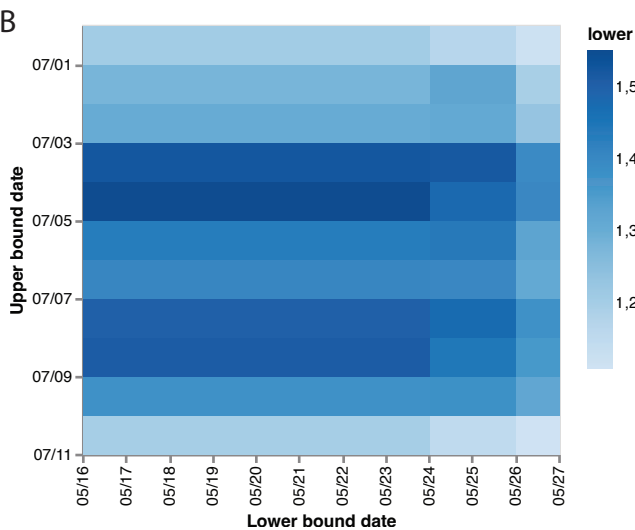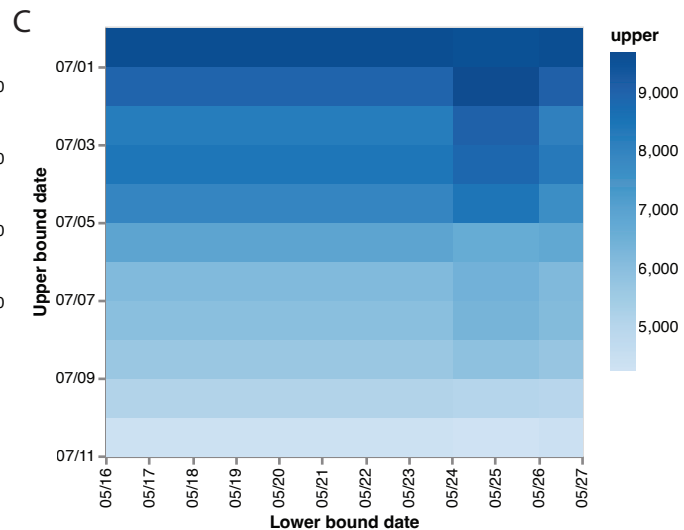

Supplement: Supplemental Information 5 — (A) the posterior median of number of cases, (B) and (C) the lower (2.5th percentile) and upper (97.5th percentile) bound of the 95% credible interval, respectively. Horizontal axis indicates the lower bound date, while the vertical axis indicates the upper bound which were used for the exponential phase window. The date is indicated as month/day in 2023. [file peerj-12-16908-s005.pdf]

A

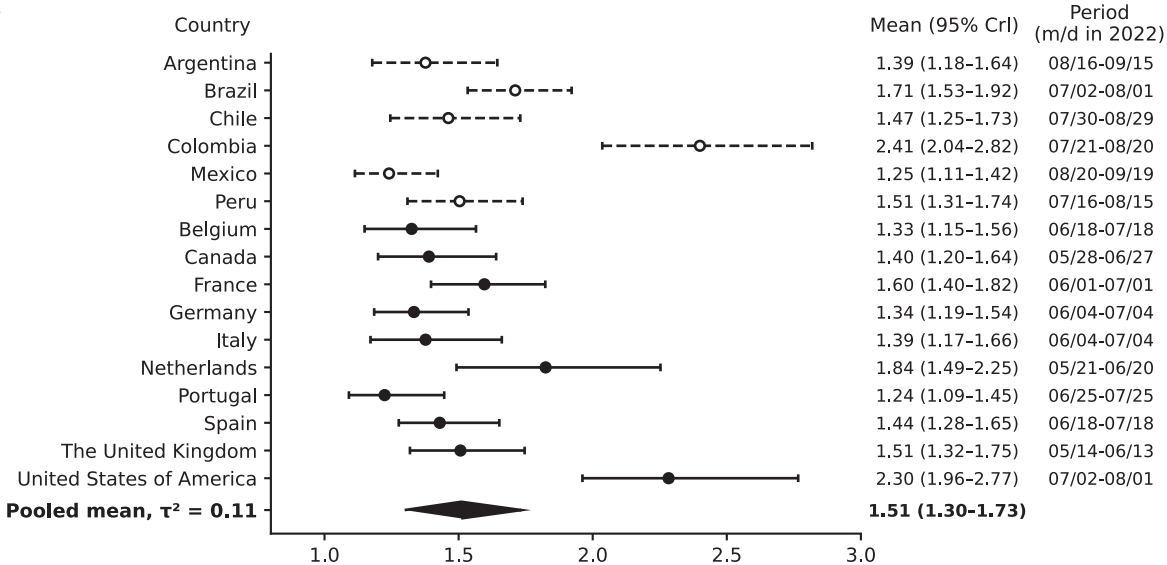

B

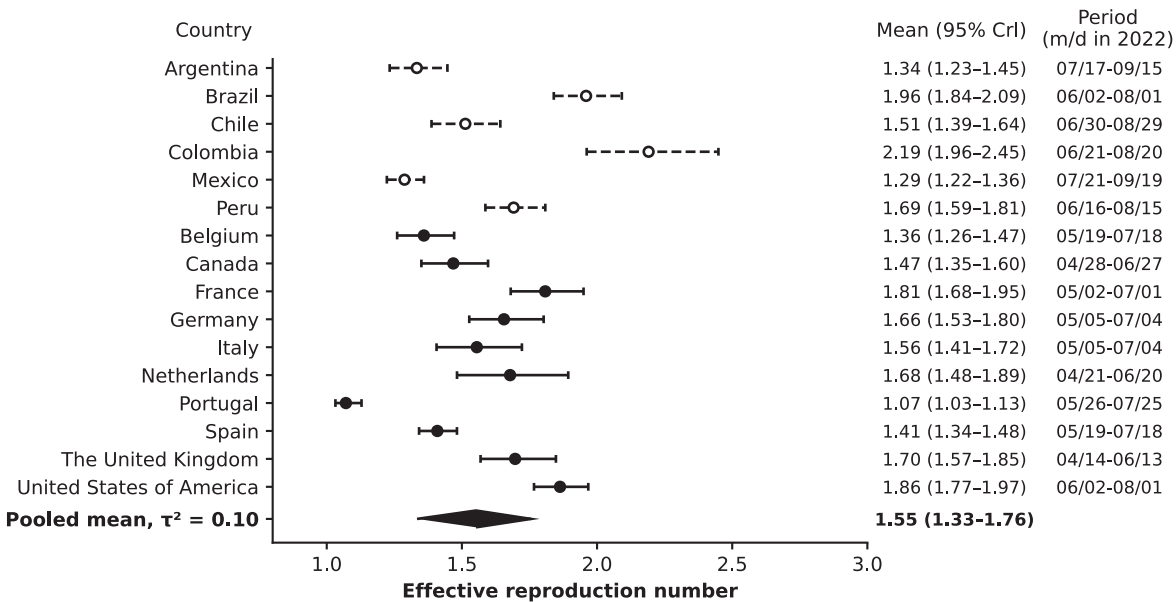

Supplement: Supplemental Information 6 — The Re values are shown in the first column on the right, while a time period of 30 days (A) or 60 days (B) is indicated in the last column. Countries that have not reported case counts, subtyped by date of symptom onset, diagnosis date, and reporting dates, were excluded from calculation of the pooled mean and are shown in dashed black. [file peerj-12-16908-s006.pdf]

# WHO China

as of 12 September 2023

**Date type**

- Diagnosis
- Reported
- Onset

Number of cases

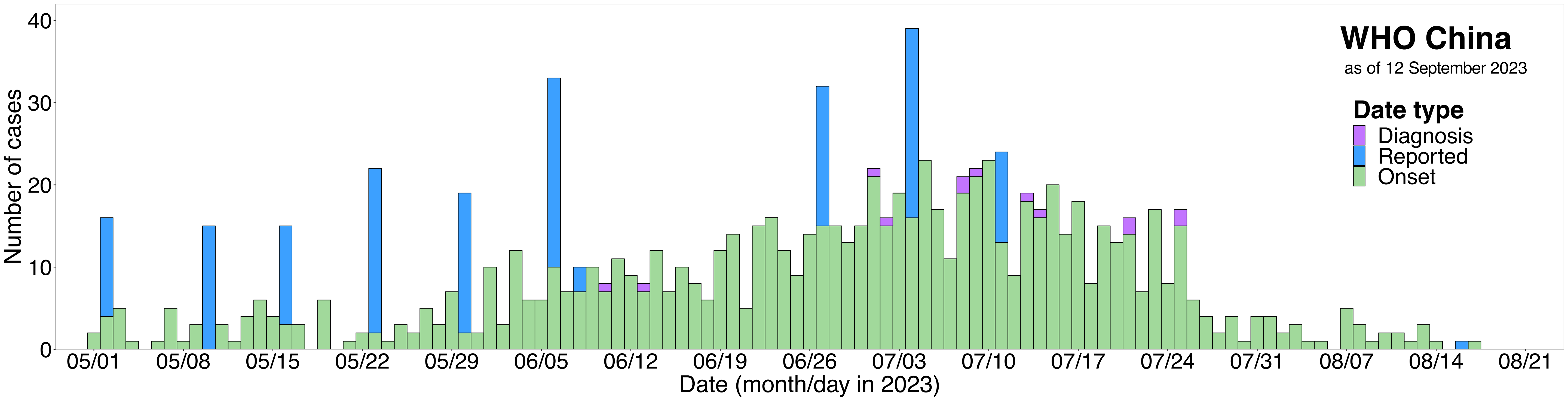

Supplement: Supplemental Information 7 — Each case count is subtyped by the date of symptom onset, the date of diagnosis, and the date of reporting. [file peerj-12-16908-s007.pdf]
